# Ceftazidime retains *in vivo* efficacy against strains of *Stenotrophomonas maltophilia* for which traditional testing predicts resistance

**DOI:** 10.1128/msphere.00840-24

**Published:** 2025-05-22

**Authors:** Matthew C. Phillips, Bosul Lee, Sarah L. Miller, Jun Yan, Kristine Goy, Marlène Maeusli, Tina Lam, Catherine Spellberg, Michael Spellberg, Rosemary She, Brad Spellberg, Brian Luna

**Affiliations:** 1 Division of Infectious Diseases, Department of Medicine, Massachusetts General Hospital 551672, Boston, Massachusetts, USA; 2 Harvard Medical School 1811 , Boston, Massachusetts, USA; 3 Department of Molecular Microbiology and Immunology, Keck School of Medicine at USC 12223, Los Angeles, California, USA; 4 Department of Pathology, City of Hope 378541 , Duarte, California, USA; 5 Los Angeles General Hospital, Los Angeles, California, USA; University of Nebraska Medical Center College of Medicine, Omaha, Nebraska, USA

**Keywords:** gram-negative bacteria, antibiotic resistance, beta-lactamases, beta-lactams, *Stenotrophomonas*

## Abstract

*Stenotrophomonas maltophilia* is responsible for a growing number of nosocomial infections and is difficult to treat owing to limited antibiotic susceptibilities. However, there are numerous recently published examples where traditional susceptibility testing methodology fails to accurately predict *in vivo* efficacy. We sought to determine if there were efficacious antibiotics against *S. maltophilia* that have been overlooked due to specious *in vivo* resistance determined by traditional *in vitro* methods. Antibiotic resistance testing was performed utilizing conventional and nutrient-limited media. Antibiotics with discordant minimum inhibitory concentrations (MICs) between the two media were selected for further experimentation. Metal ions were supplemented back into the nutrient-limited media to establish possible mechanisms. *In vivo* corroborations of *in vitro* MICs were done utilizing two infection models, *Galleria mellonella* and a neutropenic mouse oral aspiration pneumonia model. *S. maltophilia* MICs were significantly lower for ceftazidime in nutritionally deficient media that better corresponds to the *in vivo* environment than conventional rich media, resulting in a high percentage of strains determined resistant in traditional media being determined susceptible in nutritionally deficient media. The addition of zinc and manganese to the deficient media abrogated this difference, which was dependent on the L1 metallo-β-lactamase (MBL). Ceftazidime protected both *G. mellonella* and neutropenic mice against lethal infection caused by *S. maltophilia* that was predicted to be resistant in traditional media but susceptible in nutrient-deficient media. Ceftazidime may remain a viable therapeutic option for patients with *S. maltophilia* infection caused by strains predicted to be resistant by traditional susceptibility testing. Sequestration of trace metals in the host environment may prevent *S. maltophilia* MBL activity against ceftazidime.

**IMPORTANCE:**

Breakpoint interpretation criteria for ceftazidime against *S. maltophilia* were recently removed by CLSI and the FDA. It was noted that clinical data were insufficient to validate the current breakpoints. Clinical data were mixed, with some studies reporting treatment success, but others reporting treatment failure. We believe that antimicrobial testing is suboptimal, and improved testing strategies, such as the use of zinc-limited media for culture, will better model the activity of ceftazidime *in vitro*. Improved susceptibility testing strategies may better discriminate against those isolates that are truly resistant from those that were previously falsely identified as being resistant using conventional testing methods.

## Introduction

Antibiotic-resistant bacteria impose a significant burden on healthcare. In 2017, the U.S. Centers for Disease Control (CDC) estimated that there were 2.8 million infections and more than 35,000 deaths due to infections caused by antibiotic-resistant bacteria in the United States (1). These estimates are nearly double the previous estimates that were published in 2013 (1, 2). In 2019, global estimates were 4.95 million deaths associated with bacterial antimicrobial resistance (3).

*Stenotrophomonas maltophilia* is a ubiquitous, gram-negative bacillus that is responsible for an increasing number of antibiotic-resistant nosocomial infections (4, 5). *S. maltophilia* infections most commonly occur in critically ill patients, those with invasive devices such as central catheters or endotracheal tubes, and those on broad-spectrum antibiotics (6, 7). It is also commonly isolated in patients with respiratory diseases such as cystic fibrosis (8). *S. maltophilia* is notoriously difficult to treat due to an arsenal of intrinsic antibiotic resistance mechanisms including multidrug efflux pumps, membrane modifications, and antibiotic-modifying enzymes (5, 9). Notably, *Stenotrophomonas* has two chromosomally encoded β-lactamases, L1, a metallo-β-lactamase (MBL) that utilizes heavy metal ions like zinc as a cofactor to hydrolyze penicillins, cephalosporins, and carbapenems, and L2, a serine-cephalosporinase, which hydrolyzes a wider spectrum of cephalosporins as well as aztreonam (5, 7). Furthermore, minimum inhibitory concentration (MIC) data are only available for a limited number of clinically relevant antibiotics against *S. maltophilia* by the Clinical and Laboratory Standards Institute (CLSI) and the European Committee on Antimicrobial Susceptibility Testing (EUCAST) due to testing and reproducibility challenges (10, 11).

Failure to initially select an appropriate antibiotic leads to increased mortality in *S. maltophilia* infections (12). Furthermore, the intrinsic resistance of *S. maltophilia* to β-lactam antibiotics greatly limits therapeutic options for these deadly infections.

However, this resistance profile has historically been determined by susceptibility testing using nutrient-enriched growth media that poorly mimic nutrient-limited *in vivo* conditions. We recently found the mammalian cell culture medium RPMI-1640, which is relatively nutrient-depleted compared with traditional cation-adjusted Mueller-Hinton broth (CAMHB), better predicted *in vivo* efficacy of the antibiotic rifabutin against extensively drug-resistant (XDR) *Acinetobacter baumannii* (13–15). Similarly, other labs have also independently found that modified antimicrobial susceptibility testing conditions have better predicted *in vivo* outcomes for some specific drug/pathogen combinations (16–22). These results underscore the potential that growth media can alter the antibacterial effects a drug has *in vitro* compared with *in vivo* and raise the possibility that already existing antibiotics may have activity against *S. maltophilia* that has been unappreciated due to the use of nutrient-rich testing media. In this study, we expand on prior results to evaluate the accuracy of RPMI-1640 versus CAMHB in predicting *in vivo* efficacy of antibiotics against *S. maltophilia*.

## RESULTS

### MIC screening in different media conditions

**Fig 1:**
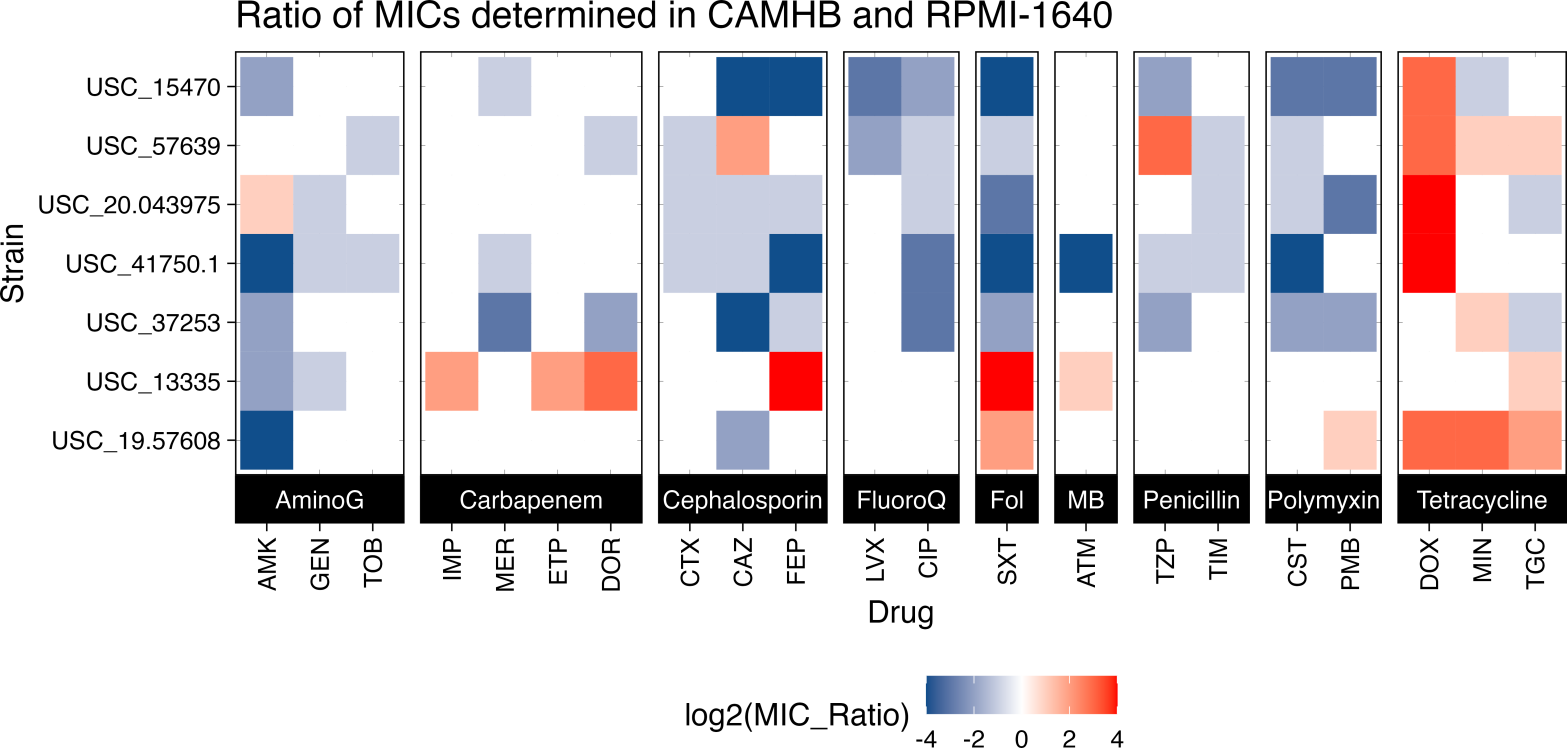
*S. maltophilia* MICs changed in different growth media. This heat map represents 294 individual MICs against a panel of *S. maltophilia* clinical isolates. MICs were determined using either CAMHB or RPMI-1640 for culture. The MIC ratio is determined by MIC_RPMI_/ MIC_CAMHB_. Amikacin (AMK), gentamicin (GEN), tobramycin (TOB), doripenem (DOR), ertapenem (ETP), imipenem (IMP), meropenem (MEM), cefepime (FEP), cefotaxime (CTX), ceftazidime (CAZ), ciprofloxacin (CIP), levofloxacin (LVX), trimethoprim/sulfamethoxazole (SXT), aztreonam (ATM), piperacillin/tazobactam (TZP), ticarcillin/clavulanic acid (TIM), colistin (CST), polymyxin B (PMB), doxycycline (DOX), minocycline (MIN), and tigecycline (TGC).

**Fig 2:**
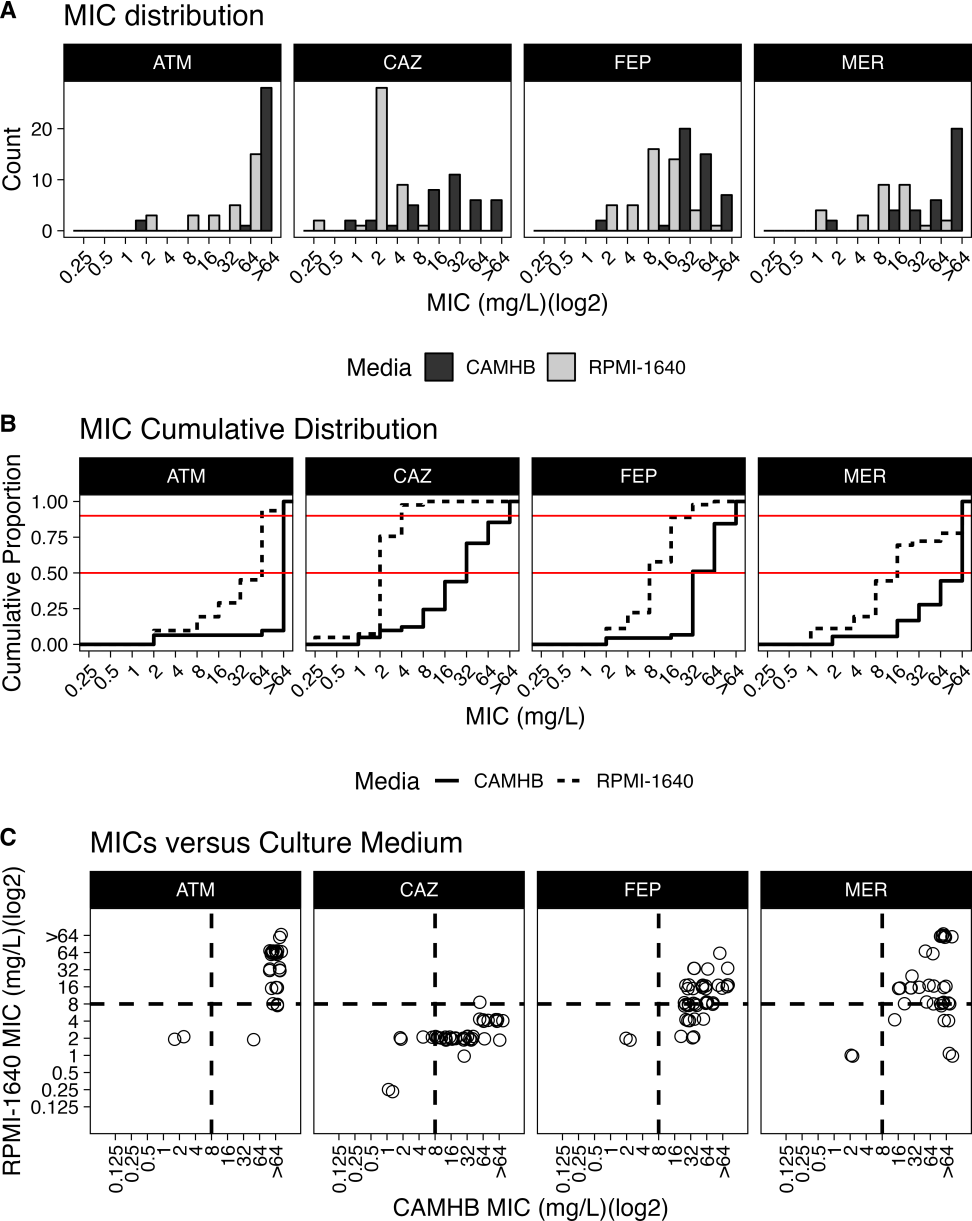
*S. maltophilia* is more susceptible to CAZ but not ATM and FEP in biologically relevant media. MICs were determined in CAMHB or RPMI-1640 media against *S. maltophilia* clinical isolates (*n* = 16). (**A**) Comparing within drugs, there was a significant difference for MICs determined in CAMHB compared with RPMI-1640 (*P* = 6.89E−9 (ATM), 1.47E−11 (FEP), 3.53E−13 (CAZ), and 5.02E−5 Mann-Whitney). (**B**) The cumulative distribution of MICs. The horizontal red lines represent the 50th and 90th percentiles. (**C**) Axes represent different growth conditions. The dashed lines indicate the susceptible breakpoints. Everything to the left of the vertical line is predicted to be susceptible in CAMHB, and everything below the horizontal line is predicted to be susceptible in RPMI-1640. Right lower quadrant represents isolates resistant in CAMHB but susceptible in RPMI-1640. CAZ was significantly different from ATM (Mann-Whitney, *P* = 7.47E−6) and FEP (Mann-Whitney, *P* = 7.41E−6). There was no significant difference between the ATM and FEP groups. All MICs determined for CAZ in RPMI-1640 were ≤8 mg/L and represent a susceptible breakpoint interpretation.

To determine whether *S. maltophilia* antibiotic susceptibility changes in biologically relevant media, we initially screened eight *S*. *maltophilia* clinical isolates for resistance against 21 antibiotics in traditional media, CAMHB, or nutrient-depleted media, RPMI-1640. A heat map was produced to compare the MIC for each *S. maltophilia* isolate in RPMI-1640 versus CAMHB (Fig. 1). Most strains demonstrated decreased MICs (increased susceptibility) in RPMI-1640 for amikacin, polymyxins, ciprofloxacin, sulfamethoxazole, and two cephalosporins: ceftazidime and cefepime. Additionally, most strains lost susceptibility to tetracyclines in the RPMI-1640, a finding previously described by other labs (16). The increased susceptibility to CAZ, and to a lesser extent FEP, in RPMI-1640 often led to strains switching from resistant to susceptible based on previous CLSI breakpoints (Fig. 2). Due to the potential clinical implications of this change, CAZ and FEP were chosen for further testing and validation utilizing the standard broth microdilution (BMD) technique commonly used in clinical microbiology laboratories. We additionally tested ATM due to its chemical similarities to CAZ.

Utilizing BMD, we next tested a panel of 14 *S*. *maltophilia* clinical isolates known to grow in RPMI-1640 media for susceptibility to CAZ, FEP, and ATM. A significant reduction in MICs was found in the nutrient-depleted, RPMI-1640 media compared with nutrient-rich, CAMHB in ATM (*P* = 6.89E−9, Mann Whitney), FEP (*P* = 1.47E−11, Mann-Whitney test), and CAZ (*P* = 3.53E−13, Mann Whitney) (Fig. 2A). All MICs determined for CAZ in RPMI-1640 were less than 8 mg/L, reflecting susceptibility by previous breakpoint interpretation. Furthermore, the shifts in antibiotic susceptibility (ratios of MIC_RPMI_/ MIC_CAMHB_) were significantly different in CAZ compared with those of both ATM (*P* = 7.47E−6, Mann Whitney) and FEP (*P* = 7.41E−6, Mann Whitney) (Fig. 2B). No significant difference was observed between the ATM and FEP groups when comparing the shift in antibiotic susceptibility across the two media conditions. CAZ was therefore selected for *in vitro* mechanistic MICs and *in vivo* validation.

To test if MICs in RPMI-1640 always resulted in susceptible CAZ MICs, we selected spontaneous mutants on RPMI-1640 agar. All isolates were categorized as CAZ-resistant or CAZ-susceptible when MICs were conducted with CAMHB or RPMI-1640, respectively. We were able to isolate CAZ-resistant mutants for seven of nine isolates (MIC > 32 mg/L) and CAZ-intermediate isolates for the remaining two of nine strains (Table 1). Therefore, the use of RPMI-1640 media for culture does not universally result in CAZ-susceptible MICs.

**TABLE 1:** Selection of CAZ-resistant mutants in RPMI-1640^*a*^

Clinical isolate	Media	Parent CAZ MIC (mg/L)	Spontaneous mutant CAZ MIC (mg/L)
46088-1	CAMHB	32	>64
46088-1	RPMI-1640	4	64
37253-2	CAMHB	16	>64
37253-2	RPMI-1640	2	16
NLF-1	CAMHB	16	>64
NLF-1	RPMI-1640	2	16
41321	CAMHB	>64	>64
41321	RPMI-1640	4	>64
41750-1	CAMHB	>64	>64
41750-1	RPMI-1640	4	32
446088-1	CAMHB	32	>64
446088-1	RPMI-1640	2	32
49468-3	CAMHB	>64	>64
49468-3	RPMI-1640	4	>64
20-050033	CAMHB	32	>64
20-050033	RPMI-1640	2	32
14570	CAMHB	64	>64
14570	RPMI-1640	2	16

^*a*^ MICs were determined by the broth microdilution method.

### Effect of cations on CAZ efficacy

Next, to determine if the difference in MICs was due to the lack of a specific nutrient in the RPMI-1640 or rather to an inhibitor in CAMHB reducing the activity of CAZ, we conducted media mixing studies. We serially mixed dilutions of RPMI-1640 with CAMHB and repeated MIC testing. Even with the smallest amount of CAMHB mixed with RPMI, the resistance pattern seen in the CAMHB media was restored. This suggested there was a nutrient missing in RPMI-1640, but present in CAMHB, that enabled *S. maltophilia* to grow despite the presence of CAZ.

**Fig 3:**
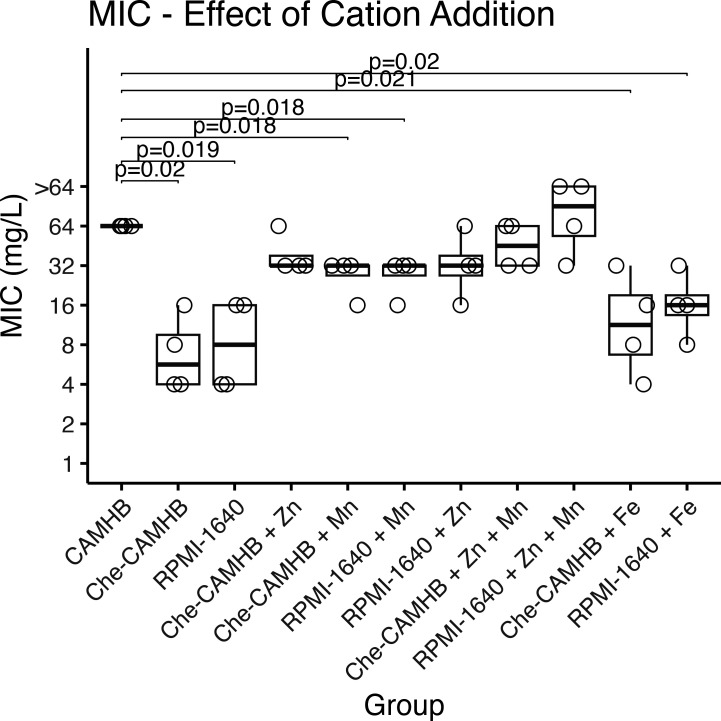
Effects of metal cations on MIC to CAZ. MICs *S. maltophilia* strain 46088–1 WT were determined using either CAMHB or RPMI-1640 or supplemented as described in the figure. “ID-CAMHB” = ion-depleted CAMHB culture. ZnCl_2_ and MnCl_2_ were supplemented at a final concentration of 10 µM, and Fe citrate was supplemented at a final concentration of 100 µM.

Although plentiful in CAMHB, there is a scarcity of metal cations in RPMI-1640, making it more closely reflective of *in vivo* conditions (13, 23, 24). Furthermore, previous research has demonstrated the necessity of metal ions, such as Zn^2+^ and Mn^2+^, for the functioning of the metallo-β-lactamases in *S. maltophilia*. We therefore hypothesized that these β-lactamases were active in nutrient-rich media but relatively less active in metal-deficient, nutrient-depleted media, leading to lower MICs in the depleted media (21, 22, 25–28). To test this hypothesis, metal cations were removed from CAMHB utilizing a metal chelator. Zn^2+^ and Mn^2+^ were then readded to this chelated-CAMHB (Che-CAMHB) as well as the RPMI-1640 to physiologic levels, and MICs were determined by BMD. Iron was added to both media to serve as a control, as it is not known to be necessary for the functioning of *S. maltophilia* β-lactamases. There was a significant reduction in MIC in Che-CAMHB, to levels similar to RPMI-1640 media, compared with standard CAMHB (*P* = 0.02). This reduction in MIC was partially ameliorated with the reintroduction of either Zn^2+^ or Mn^2+^ to the Che-CAMHB media but was not observed with iron supplementation. Following the addition of both Mn^2+^ and Zn^2+^, MICs from both the Che-CAMHB and RPMI-1640 increased to similar levels as with the CAMHB. This effect was not seen after the addition of iron to either medium (Fig. 3).

### Effect of cations on *L1-* and *L2*-mediated resistance

We first cloned and recombinantly expressed *S. maltophilia bla_L1_* but found that the gene was poorly expressed (Fig. S1). We then obtained *E. coli* MC1000 that recombinantly expressed *S. maltophilia* genes *bla_L1_* and *bla_L2_* as a kind gift from the Mavridou lab (29). We found that the abundance of zinc only affected resistance in *E. coli* strains expressing the L1 MBL enzyme but did not affect resistance mediated by the L2 serine-β-lactamase (Table 2).

**TABLE 2:** CAZ resistance mediated by L1, but not L2, is affected by cations^*a*^

Strain	CAZ MIC (mg/L)	
	CAMHB	CAMHB + 30 mg/L EDTA
*E. coli* MC1000	≤0.25	0.5
*E. coli* MC1000::pDM1	≤0.25	≤0.25
*E. coli* MC1000::pDM1_L1	128	≤0.25
*E. coli* MC1000::pDM1_L2	32	32
*S. maltophilia* 910 WT	64	4

^*a*^ The *S. maltophilia* genes *blaL1* and *blaL2* were recombinantly expressed in *E. coli* MC1000 (29). In the presence of the cation-chelating agent EDTA, *E. coli* expressing the *blaL1* became susceptible to CAZ. However, the presence of EDTA did not affect *E. coli* that was expressing the serine β-lactamase *blaL2*.

### CAZ efficacy in *Galleria mellonella*

Given the difference in *in vitro* activity in nutrient-rich vs. -depleted media, we sought to determine which better predicted *in vivo* efficacy. *G. mellonella* was initially chosen due to its widespread use in studying host-pathogen interactions with *S. maltophilia* (30–32). First, we established the inoculum necessary to kill *G. mellonella* larvae over 1–4 days using a WT strain of *S. maltophilia* that is resistant to CAZ by standard CAMHB methods but susceptible when tested in RPMI-1640 media. Utilizing this same strain, *G. mellonella* was infected with a lethal inoculum and subsequently treated with a single dose of either 5, 15, or 50 mg/kg of CAZ or PBS as a control. At doses down to 15 mg/kg, CAZ was able to rescue *G. mellonella* against a resistant strain of *S. maltophilia* (*P* = 3.6E−5; log-rank test). These data, although in conflict with the CAMHB-determined susceptibilities, are consistent with the results obtained utilizing the RPMI-1640 media.

**Fig 4:**
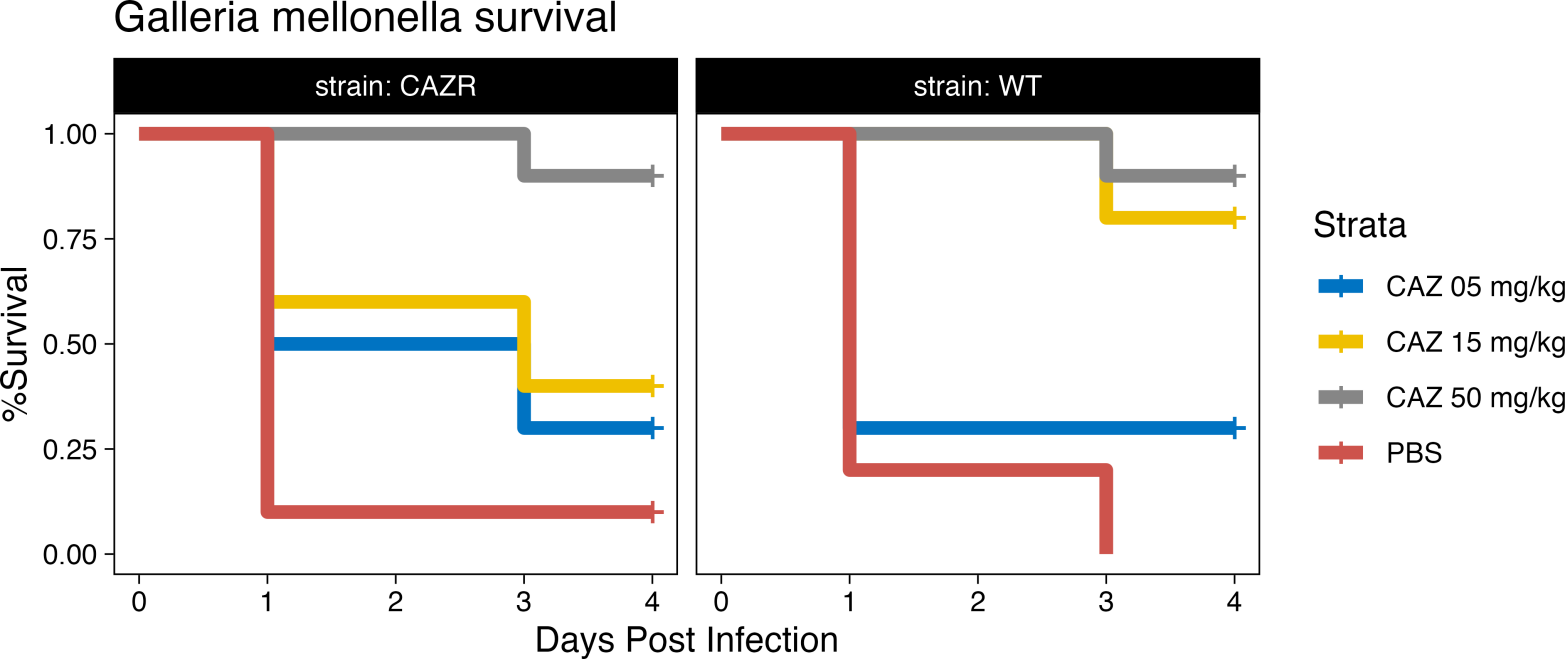
CAZ efficacy in a *G. mellonella* infection model. Larvae were challenged with 1.1E7 or 9.6E6 CFUs of *S. maltophilia* 46088-1 WT or CazR, respectively, and then treated with PBS or CAZ. No infection controls received two doses of PBS. Treatment with 50 mg/kg CAZ was capable of rescuing both WT- and CazR-infected larvae in a statistically significant manner (log-rank test, *P* < 0.0001 or =0.0002, respectively). Treatment with 15 mg/kg CAZ, however, was only able to significantly rescue WT-infected larvae (log-rank test, *P* < 0.0001 versus *P* = 0.0614).

**Fig 5:**
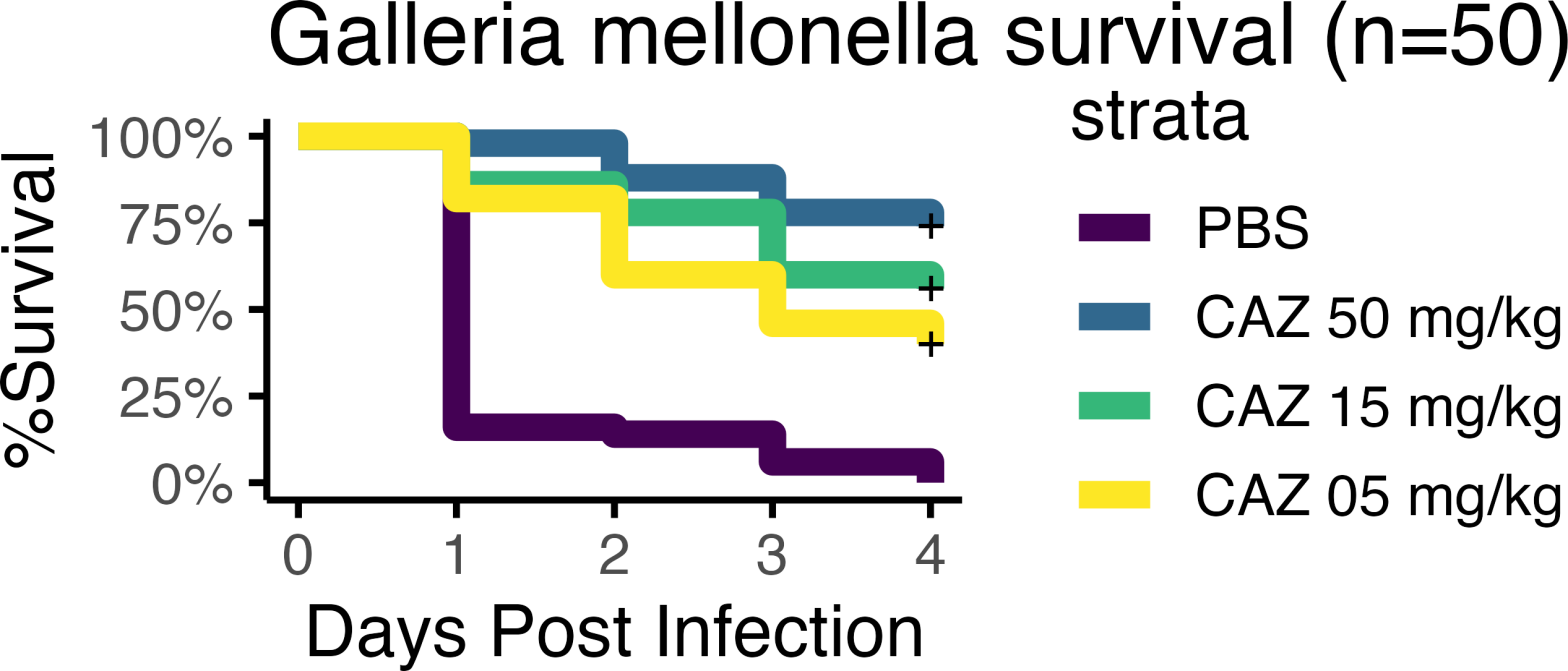
CAZ efficacy in a *G. mellonella* infection model. Larvae (*n* = 10) were challenged with five different *S. maltophilia* clinical isolates that were susceptible as defined by MICs in Che-CAMHB and then treated with PBS or CAZ, and all data were combined (*n* = 50 per group). Treatment with 50 mg/kg CAZ rescued 39 (78%) of infected larvae (log-rank test, *P* < 0.0001).

To further test the biologic relevance of susceptibilities determined in this media, we developed a strain of *S. maltophilia,* which was resistant to CAZ even in the low cation media, with the hypothesis that CAZ would fail to rescue the *G. mellonella* from infection with this CAZ-R strain. Consistent with the results in the RPMI-1640 media, CAZ was unable to rescue the larva at doses of 15 mg/kg, although this could be overcome with higher doses of 50 mg/kg (*P* = 1.8E−4; log-rank test) (Fig. 4). To further support that this treatment efficacy was reproducible, we repeated the *G. mellonella* infection and treatment experiments using five different clinical isolates that were predicted to be susceptible in Che-CAMHB. We found that treatment efficacy was generally reproducible, and treatment with 50 mg/kg CAZ rescued 39 (78%) of infected worms compared with 3 (6%) of the PBS treatment group (*P* < 0.001; log-rank test) (Fig. 5).

### CAZ efficacy in murine oral aspiration (OA) model

**Fig 6:**
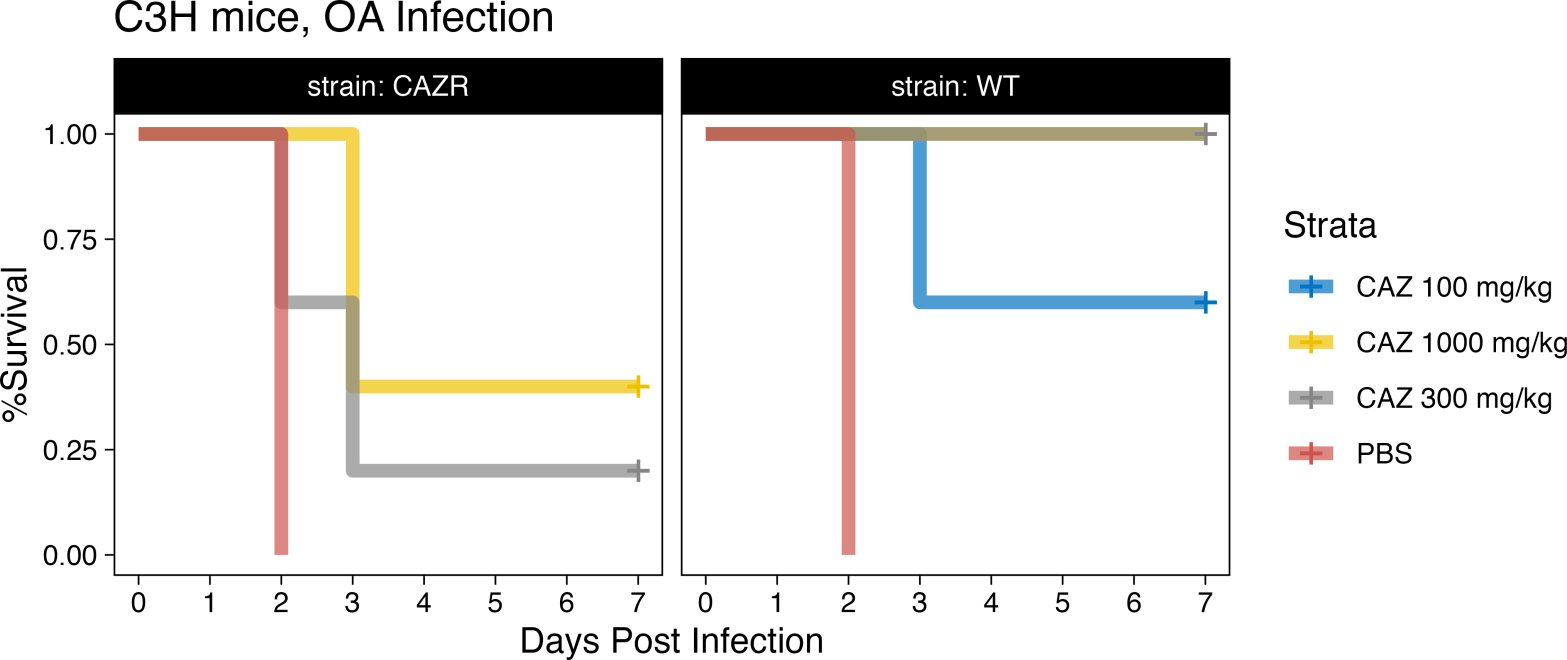
CAZ efficacy in a murine infection model. Dosing and dosing intervals for a 100 mg/kg were done as previously described (33). The dosing interval was kept constant, and doses administered were proportionally scaled up for the 300 mg/kg and 1,000 mg/kg treatment groups. The CAZ 300 and CAZ 1000 treatment groups overlap in the WT panel.

To further validate the *in vivo* efficacy of CAZ against *S. maltophilia* in respiratory disease, we determined murine C3HeB/FeJ survival in an oral aspiration pneumonia infection model. This model is meant to simulate hospital-acquired pneumonia, specifically ventilator-associated pneumonia, the most common type of infection seen with *S. maltophilia* (7). We inoculated neutropenic mice with a lethal dose of the same *S. maltophilia* strains tested in the *G. mellonella* model (one of which was susceptible in RPMI-1640 media and one of which was resistant in RPMI-1640, but both of which were resistant in traditional rich media). Mice were then treated for 3 days with 100, 300, or 1,000 mg/kg of CAZ or PBS as a control. Similar to what was seen in the *G. mellonella* model, CAZ was able to rescue mice infected with the strain susceptible in RPMI-1640 media at all three doses, including the lowest, 100 mg/kg (*P* = 1.62E−2; log-rank test with Bonferroni correction). When mice were infected with the strain resistant in RPMI-1640, the 300 mg/kg dose of CAZ was unable to rescue the mice. This resistance was able to be overcome with the highest dose (1,000 mg/kg), however (*P* = 0.014; log-rank test with Bonferroni correction) (Fig. 6).

## DISCUSSION

CAZ is not recommended for use against *S. maltophilia* due to increasing resistance, with the percentage of susceptible *S. maltophilia* isolates decreasing from up to 75% in the late 90s to between 30.5% and 36.8% between 2009 and 2012 (5). Recent treatment guidelines issued by the IDSA specifically recommend *against* the use of CAZ for the treatment of *S. maltophilia*, going as far as to say clinical microbiology laboratories and antibiotic stewardship teams should convey the likely ineffectiveness of CAZ, which recently was removed from CLSI published breakpoints for *S. maltophilia* (10). These recommendations, however, are all based on current standard testing protocols and *in vitro* experiments that rely on nutrient-enriched CAMHB media, which may not predict true *in vivo* efficacy (13–16, 28, 34–39). Our studies confirm that traditional testing mischaracterizes strains as resistant when they are actually susceptible to CAZ in nutritionally depleted media *in vitro* and effective in treating infection *in vivo*. These results suggest that CAZ may remain an effective therapeutic option and that clinical testing is warranted to validate these pre-clinical findings.

Interestingly, of the drugs screened, many initially CAZ-resistant strains of *S. maltophilia* in CAMHB showed dramatic decreases in their MIC when tested in the RPMI-1640 media (Fig. 1). When testing for CAZ susceptibility on a wider array of *S. maltophilia* strains, utilizing the standard CLSI methods for BMD, all strains consistently had lower MICs when tested in the media more closely simulating *in vivo* conditions (Fig. 2). The biological relevance of these *in vitro* results was validated using two infection models, *G. mellonella* and a mouse model of ventilator-associated pneumonia. In both models, animals survived what should have been a lethal infection by receiving CAZ, an ostensibly ineffective drug based on traditional MIC testing (Fig. 4 to 6). Importantly, when a CAZ-resistant strain was generated utilizing RPMI-1640 media, the biologically relevant media correctly predicted the lack of efficacy of CAZ *in vivo* as well. In both cases, the clinical decision to use CAZ would change based on the testing media used.

One unique challenge pathogens encounter during an infection is a paucity of nutrients such as metal cations, which are intentionally sequestered by the host organism (40–42). Our data show that the removal of metal ions from traditional CAMHB media recreates MICs comparable with those obtained from the physiologically relevant media. Similar to prior work on the L1 MBL in *S. maltophilia*, the addition of Zn^2+^ and Mn^2+^ to the media allows the bacteria to regain its resistance to ceftazidime (Fig. 3) (25, 26). We further show that this MIC discordance can be recreated in *E. coli* by the addition of this MBL enzyme but not by the addition of the S. maltophilia L2 serine ꞵ-lactamase. Given that the activity of the L2 enzyme is independent of metal ions, this is consistent with the failure to restore susceptibility to cefepime and aztreonam in metal-depleted media, both of which are better substrates to the L2 serine ꞵ-lactamase than the L1 MBL like CAZ (43). Taken together, this suggests that resistance to CAZ, as measured in traditional media, is mediated by the L1 MBL utilizing excesses of heavy metal ions like Zn^2+^ and Mn^2+^, which leads to an apparent resistance to this antibiotic. As demonstrated by our *in vivo* experiments, though, this resistance is an artifact of the excess nutrients available in traditional media and does not reflect the ability of these bacteria to withstand this antibiotic in an infected host sequestering the necessary metal ions for effective activity of the L1 MBL. Similar metal ion-dependent discordance between *in vitro* resistance and *in vivo* susceptibility has been shown in MBL-producing strains of *K. pneumonia* and other *Enterobacterales* after exposure to carbapenems, ceftazidime, cefepime, and aztreonam (21, 22, 27, 34, 44, 45).

These data raise the possibility that *in vitro* resistance mediated by MBLs, as determined in standard CAMHB, may have limited utility in ascertaining the *in vivo* effectiveness of these drugs in a metal-depleted environment. Given this potential, there is a need for more work to be done looking at existing antibiotics, particularly those traditionally thought to be deactivated by metallo-β-lactamases, as potential new therapies for multidrug-resistant organisms such as *S. maltophilia*.

In summary, CAZ may remain an important therapeutic option for the treatment of *S. maltophilia,* particularly for strains resistant to other antibiotics, even when traditional laboratory testing suggests high MICs in rich media. These results also call into question the accuracy of susceptibility testing for organisms containing metallo-β-lactamases when conducted in nutrient-rich media and reiterate the need for clinical validation of *in vitro* susceptibility testing results, particularly if such results are going to be used to establish a standard of care for infections with limited therapeutic options.

## MATERIALS AND METHODS

### Strain selection and preparation

Wild-type (WT) *S. maltophilia* strains used for experiments were clinical isolates (Keck Medical Center of USC, Los Angeles, CA). Spontaneous ceftazidime-resistant *S. maltophilia* mutants were developed by serial passaging under antibiotic selection. *S. maltophilia* 46088-1 wild type was grown overnight in tryptic soy broth (TSB) at 37°C/200 rpm. The overnight culture was centrifuged at 4,000 rpm for 5 min, washed 1× with PBS, and resuspended in 1 mL PBS. An inoculation loop was used to streak this bacterial suspension on gradient RPMI-1640 agar plates containing 0 mg/L to 32 mg/L CAZ, 2.05 mM glutamine, and 10% FBS (46). After incubation at 37°C for 48 h, aliquots of RPMI-1640 supplemented with 2.05 mM glutamine, 10% FBS, and 8 mg/L CAZ were inoculated with single mutant colonies and grown overnight at 37°C/200 rpm. Each subsequent day, 100 µL of these overnight cultures were subcultured overnight in fresh RPMI-1640 supplemented with 2.05 mM glutamine, 10% FBS, and a 2-fold increase from the day before of CAZ at 37°C / 200 rpm until cultures grew under 32 mg/L CAZ selection, which is the resistant breakpoint as defined by CLSI (47, 48). MICs were determined via broth microdilution, and one CAZ-resistant culture (>64 mg/L in CAMHB, 64 mg/L in RPMI), referred to as “*S. maltophilia* 46088-1 CazR” throughout the paper, was chosen at random and used for subsequent experiments.

*S. maltophilia* strains were grown overnight in TSB (VWR International) at 37°C/200 rpm. Overnight cultures were then diluted 1:100 and subcultured in fresh TSB until mid-log phase. Bacterial suspensions were centrifuged at 4,000 rpm for 5 min, washed 3 times with PBS, and adjusted to an optical density (OD) of 0.5 before dilution to the desired inoculum. Delivered inoculum densities were calculated by plating serial dilutions on tryptic soy agar (TSA; VWR International) plates, incubating overnight at 37°C, and enumerating colonies.

*E. coli* MC1000 isolates expressing blaL1 or blaL2 were a kind gift from the Mavridou lab (29). The gene inserts were cloned into plasmid pDM1 under the control of the Ptac promoter, and expression was induced with 0.25 mM IPTG as previously described (29).

### Sensititre MICs

Sensititre gram-negative GNX2F AST Plate (GNX2F; Thermo Scientific) was used for the initial screening of antibiotic susceptibility in both the RPMI-1640 and CAMHB. Assays were conducted based on manufacturer instructions. Briefly, overnight cultures of *S. maltophilia* were subcultured and subsequently centrifuged, washed with PBS, and adjusted to a 0.5 McFarland standard. This suspension was diluted, and 5 × 105 CFU/ml were then added to either CAMHB or RPMI-1640 media and used to inoculate the Sensititre plate. Plates were incubated for 18–24 h at 37°C and manually read and MICs recorded.

### MIC assay

Unless otherwise indicated, the broth microdilution method was used to determine MICs (13, 49). For rigor, assays were done independently in triplicate. The medium used for the MIC assays performed in the present study was either CAMHB (212322; BD Biosciences) alone or RPMI-1640 (11875119; Thermo Fisher) supplemented with 10% fetal bovine serum (PS-100, Phoenix Scientific). The final drug concentrations in the plate were 2-fold dilutions ranging from 1 to 32 mg/L. MIC inoculum concentration was confirmed by plating serial dilutions on TSA plates, incubating at 37°C overnight, and enumerating colonies. MIC plates were incubated at 35 ± 2°C without shaking, and the results were recorded at the CLSI recommended time points (49).

### Metal cation depletion and supplementation

Chelated, cation-depleted CAMHB (Che-CAMHB) was prepared based on previously validated methods (50). Briefly, Chelex 100 resin (Bio-Rad Laboratories, Hercules, CA) was added to autoclaved CAMHB in a 1:10 ratio based on weight and stirred at room temperature for 2 h. Following incubation, the media was run through a 0.2 µm filter to remove the resin and ensure sterility. Manganese chloride (Sigma, M1787-100ML), zinc chloride (Sigma, 39059-100ML-F), and ferrous citrate (Sigma, F3388-250G) stocks were prepared in sterile water and then added back to this ion-depleted media or RPMI-1640 for MIC testing.

### *Galleria* model

*G. mellonella* larvae (WAXB500; Timberline) were stored at room temperature in the dark for no longer than 1 week before experimentation. Larvae weighing 170–350 mg and grouped by tens were incubated at 4°C for up to 1 h before infection to reduce their movement during injection. Bacteria were prepared for infection, and CFUs delivered were enumerated as previously described. Each larva was disinfected by brief rolling in a 70% ethanol-soaked KimWipe (470224-038; Kimtech Science), restrained with a previously described restraint device (51), and infected subcutaneously through its most posterior proleg(s) using an NE-1000 fully programmable single-syringe pump (New Era Pump Systems, Inc.) with 10 µL *S. maltophilia* 46088-1 WT or CazR to determine the respective 100% lethal doses (LD100). No infection control larvae received 10 µL PBS. Larvae were then infected with previously determined LD100 (in CFU) of each strain and treated with 10 µL PBS or 5, 15, or 50 mg/kg CAZ 1 h post-infection. No infection control larvae received two doses of 10 µL PBS. For all experiments, larvae were incubated in 100 mm petri dishes (25384-302; VWR) at 37°C, and survival was monitored up to day 4 post-infection.

### Mouse studies

Fresh *S. maltophilia* 46088-1 was prepared as described above and diluted in PBS to adjust the bacterial density as needed for infection. Male C3HeB/FeJ mice (*n* = 5 per group), 9–10 weeks old, were given 150 mg/kg cyclophosphamide 4 and 1 day(s) before infection. Each mouse was infected with 2 × 107 CFUs of *S. maltophilia* 46088-1 wild type (WT) or 5 × 107 CFUs of CAZ-resistant *S. maltophilia* (CazR) via oral aspiration (52. The inoculum bacterial density was confirmed by plating serial dilutions on TSA plates and incubating overnight at 37°C.

### Antibiotic treatments

Pharmacy grade ceftazidime (NDC #0409-5084-13; Hospira) was stored at room temperature and reconstituted in sterile PBS each day. Mice were weighed to determine an average weight for dosing. Ceftazidime (CAZ) was administered to mice at 100 mg/kg, 300 mg/kg, or 1,000 mg/kg (1×, 3×, or 10×, respectively) according to the dosing strategy as previously described (33). CAZ was administered subcutaneously with 500 µL of each drug concentration three times per day for 3 days. Doses were given at 0, 3, and 6 h post-infection on day one, then at the same time points, 24 h later, for the following 2 days to complete the 3-day course of antibiotics.

### Statistics

Statistics were performed using R. Pairwise comparisons were evaluated using the Mann Whitney test. Time to death was compared using the log rank test. *P* values < 0.05 were considered significant.

## SUPPLEMENTAL MATERIAL

Fig. S1 msphere.00840-24-s0001.pdf Expression of *S. maltophilia* strain 910 in *E. coli* DH5ɑ.

ASM does not own the copyrights to Supplemental Material that may be linked to, or accessed through, an article. The authors have granted ASM a non-exclusive, world-wide license to publish the Supplemental Material files. Please contact the corresponding author directly for reuse.
