# Supplementary material for: Ceftazidime retains in vivo efficacy against strains of Stenotrophomonas maltophilia for which traditional testing predicts resistance
Source: mSphere. 2025 May 22;10(6):e00840-24. doi: 10.1128/msphere.00840-24 (PMC12188716; doi:10.1128/msphere.00840-24)
Supplement: Fig. S1 — Expression of S. maltophilia strain 910 in E. coli DH5ɑ. [file msphere.00840-24-s0001.pdf]

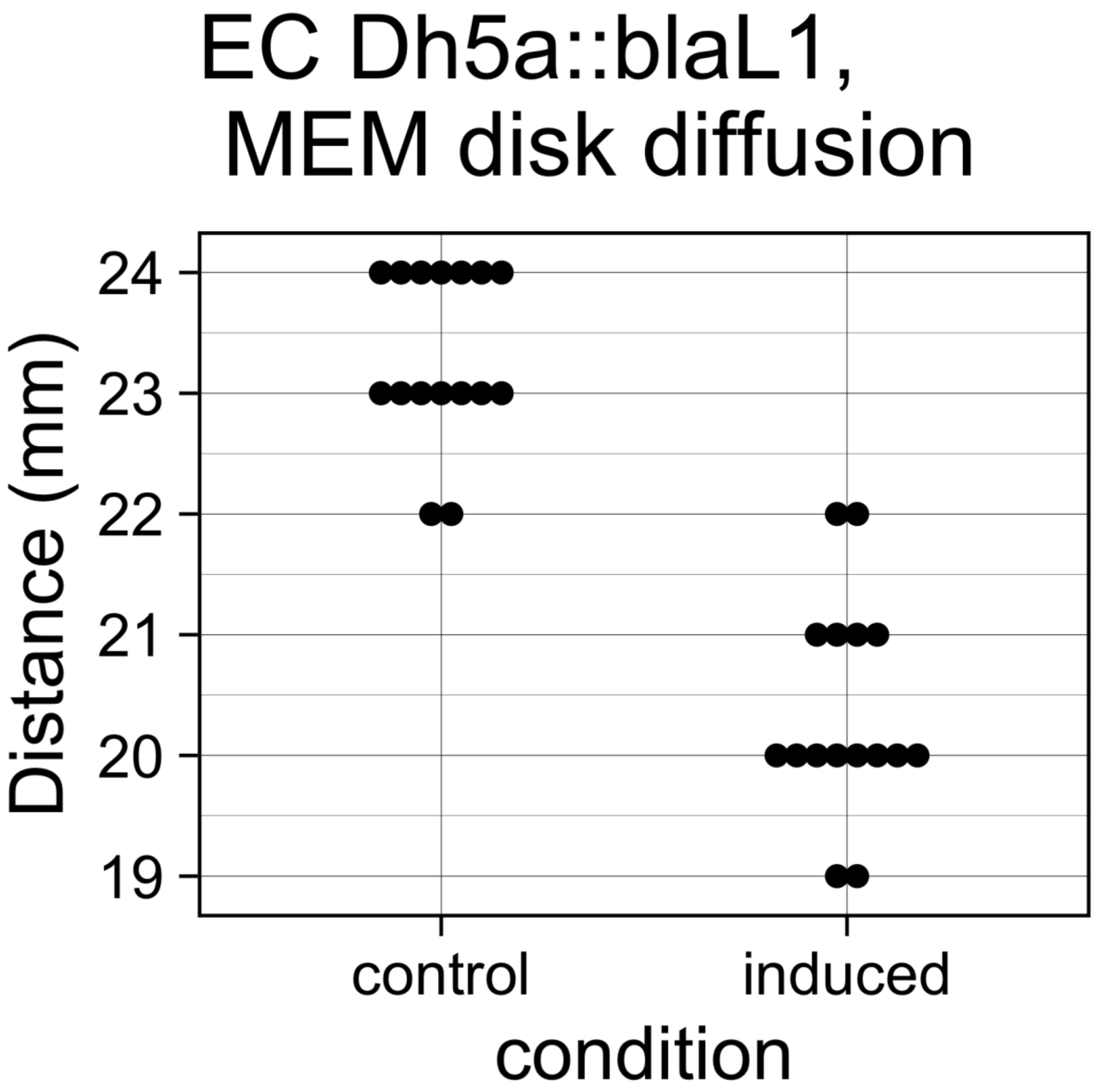

Fig. S1 Expression of *S. maltophilia* strain 910 in *E. coli* DH5α. The open reading frame of *blaL1* was cloned into pUC18T-mini-Tn7T-Apr-LAC (addgene 64965) and recombinantly expressed in *E. coli* with 10 µg of IPTG. Expression *blaL1* resulted in increased resistance to MEM (Mann Whitney, $p < 0.001$).
